# Minimal Functional Sites in Metalloproteins and Their Usage in Structural Bioinformatics

**DOI:** 10.3390/ijms17050671

**Published:** 2016-05-04

**Authors:** Antonio Rosato, Yana Valasatava, Claudia Andreini

**Affiliations:** 1 Magnetic Resonance Center (CERM), University of Florence, Via L. Sacconi 6, 50019 Sesto Fiorentino, Italy; yana.valasatava@gmail.com (Y.V.); andreini@cerm.unifi.it (C.A.); 2 Department of Chemistry, University of Florence, Via della Lastruccia 3, 50019 Sesto Fiorentino, Italy

**Keywords:** metalloenzyme, bioinorganic chemistry, structural biology, cytochrome, heme, zinc, iron

## Abstract

Metal ions play a functional role in numerous biochemical processes and cellular pathways. Indeed, about 40% of all enzymes of known 3D structure require a metal ion to be able to perform catalysis. The interactions of the metals with the macromolecular framework determine their chemical properties and reactivity. The relevant interactions involve both the coordination sphere of the metal ion and the more distant interactions of the so-called second sphere, *i.e.*, the non-bonded interactions between the macromolecule and the residues coordinating the metal (metal ligands). The metal ligands and the residues in their close spatial proximity define what we call a minimal functional site (MFS). MFSs can be automatically extracted from the 3D structures of metal-binding biological macromolecules deposited in the Protein Data Bank (PDB). They are 3D templates that describe the local environment around a metal ion or metal cofactor and do not depend on the overall macromolecular structure. MFSs provide a different view on metal-binding proteins and nucleic acids, completely focused on the metal. Here we present different protocols and tools based upon the concept of MFS to obtain deeper insight into the structural and functional properties of metal-binding macromolecules. We also show that structure conservation of MFSs in metalloproteins relates to local sequence similarity more strongly than to overall protein similarity.

## 1. Introduction

Life originated and developed on the Earth’s crust, *i.e.*, within an inorganic environment. Consequently, organisms have recruited many different metals, such as iron or zinc, for the catalysis of a significant variety of biochemical reactions. Several metals remain essential to life in extant organisms and play a diversity of roles in many different physiological processes. On the other hand, metals such as mercury or lead are poisonous to living organisms. Sometimes, the toxicity of a given element may vary significantly because of speciation, so that different chemical species containing the same metal may have a very different impact on living organisms. Bioinorganic or biological inorganic chemistry studies the interaction between inorganic substances and biological molecules [1,2]. This discipline encompasses a wide range of chemical and biological topics. Indeed, it addresses the role, uptake, and fate of essential elements, as well as the response of living organisms to toxic inorganic substances. Bioinorganic chemistry addresses also the uses of metal ions in medicine, namely applications such as the design and functional characterization of metal-based drugs, and the production of MRI contrast agents. Finally, topics of bioinorganic chemistry that are closer to its inorganic chemistry aspects are, for example, the synthetic production of functional models of metal-containing enzymes and the development of spectroscopic tools and theoretical models to support all the above topics, *etc.*

Many proteins require metal ions to carry out their physiological functions and hence are called metalloproteins or metal-binding proteins. Indeed, metalloenzymes comprise about 40% of all enzymes with known 3D structures [3]. Structural biology and structural genomics techniques can provide direct information on the chemical environment of metal ions in metalloproteins, and especially on their coordination by the protein frame [4,5,6]. Unfortunately, it is often difficult to determine experimentally which metal cofactors bind *in vivo* to a (putative) metalloprotein, because this requires elucidation of protein interactions with non-covalently bound metal ions. An answer to this issue could be provided by metalloproteomics techniques, namely a combined portfolio of analytical approaches for identification and quantification of metalloproteins in biological systems at the level of the entire proteome [7,8,9].

Metal ions are bound to biological macromolecules via coordination bonds. The coordination bonds are formed by a metal ion and the donor atoms provided by the macromolecule (protein or nucleic acid). Both the backbone and the side chains/bases of the macromolecule can provide donor atoms. Non-macromolecular ligands, such as oligopeptides, in addition to small organic molecules, anions, and water molecules can provide additional donor atoms. The metal ion (or cluster of metal ions) and its donor atoms constitute the metal-binding site. However, the mere investigation of the structural features of metal-binding sites often does not afford a satisfactory comprehension of the biochemical properties of metal sites. To achieve this goal, it is necessary to enlarge the analysis by taking into account the nearby macromolecular environment [10,11,12,13,14,15]. This larger ensemble of atoms constitutes the minimal environment determining metal function, *i.e.*, the “minimal functional site” (MFS, see next section). The MFS describes the local 3D environment around the cofactor, and it is independent of the structural properties of the protein fold binding it. In previous work, we have shown that MFSs can provide an unbiased insight into the function or mechanism of action of a metalloprotein [12,15,16,17]. This contribution focuses on the use of MFSs in structural bioinformatics of metalloproteins, by exploiting a portfolio of software tools and resources that we have developed in the past years.

## 2. The Minimal Functional Site (MFS)

**Figure 1:**
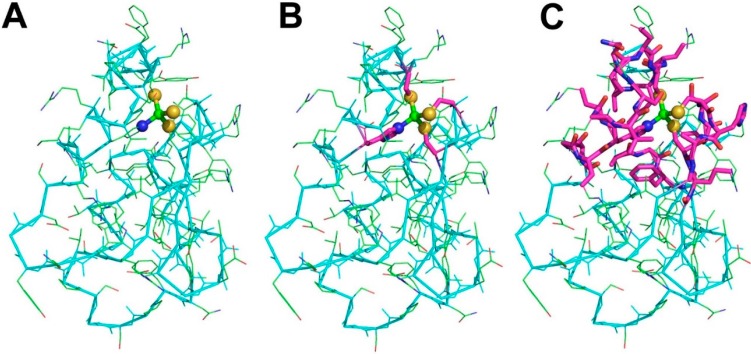
Construction of a Minimal Functional Site (MFS). (**A**) The metal (green sphere) and the donor atoms (blue and gold spheres, for the Nε2 of a His and the Sγ of three Cys); (**B**) the metal-binding site, obtained by including all the atoms of the residues providing the donor atoms (metal ligands, shown as sticks, colored magenta for carbon atoms, blue for nitrogen atoms, red for oxygen atoms, yellow for sulfur atoms); (**C**) the MFS, obtained by additionally including all protein residues with at least one atom within 5 Å from a metal ligand (sticks, same color code as in (**B**)). In all panels, the Cα trace of the protein backbone is shown as thick cyan sticks, whereas all bonds are shown as thin sticks with the same color code as panel (**B**), except for carbon atoms (green). Note that the two Cys on the right side of the metal ion are in a fragment that is separate from the rest of the MFS. The MBP of this site is CX(2)CX(16)HX(6)C.

To build a MFS we start from the identification of the metal-binding site (Figure 1A). X-ray crystallography and X-ray absorption spectroscopy are the main techniques for the detailed characterization of metal-binding sites [6,18,19,20]. Databases reporting on the geometric properties of metal-binding sites in proteins [21,22] or nucleic acids [23] are available. The information contained in such databases is computed from the contents of another database, the Protein Data Bank [24] (PDB). The PDB contains the atomic coordinates of all biological macromolecules of known 3D structure and can be considered the mother of all structural databases in biology. Some caveats are present in the literature regarding how to correctly extract metal-binding sites and MBPs from PDB structures, such as the omission of symmetry-related ligands [25]. Errors in the deposited structures can also occur, which should be remediated by reinterpretation of the electron density maps [26].

It is common practice to extend metal-binding sites in order to include all of the atoms in the amino acids or nucleotides containing the donor atoms (Figure 1B). In proteins, the identity and spacing along the sequence of the amino acid ligands define the metal-binding pattern (MBP) of the metal-binding site [27,28,29]. For example, a common MBP in zinc fingers is CX(2)CX(12)HX(2)H, where X denotes any amino acid. MBPs are extremely useful to identify metalloproteins within whole-proteome sequences [29,30,31,32,33,34]. The presence of metal sites in biological macromolecules confers specific functional properties to the system that the knowledge of the first coordination sphere alone cannot recapitulate [35,36,37]. For example, models of metal sites in proteins including only the metal ligands may not reproduce the biochemical properties of the system adequately. By taking into account the surroundings of the metal-binding site, the relationship with functional properties becomes more evident. This larger structural environment constitutes the minimal set of atoms, extracted from the 3D structure, that determine metal function. In previous work we dubbed this ensemble of atoms the Minimal Functional Site (MFS) [17]. The definition of MFS in a metal-macromolecule adduct is as follows: the MFS is the ensemble of atoms formed by the metal ion or cofactor, all its ligands (thus the metal-binding site, up to here) and any other residue or chemical species having at least one atom within 5.0 Å from a metal ligand (Figure 1C). MFSs describe the local structural environment around a metal ion or metal cofactor and do not depend on the overall macromolecular structure. Consequently, the analysis of a group of MFSs is independent of and complementary to the global fold analysis of the metalloproteins containing those sites. Importantly, the definition of MFS is equally valid for sites contained in proteins or nucleic acids as well as for sites formed at the interface between two (or more) macromolecules.

MFSs typically do not correspond to continuous stretches of the macromolecule sequence. In fact, each MFS is a group of various sequence fragments. The size of each fragment depends largely on the number of ligands it contains. With respect to the notion of MBP that we introduced before, each fragment will contain a part of the entire MBP associated with the entire MFS. The fragmented nature of MFSs makes it difficult to use standard software for their sequence or structural comparison. This was one of the reasons that warranted the development of a specialized tool, MetalS^2^, for the structural superposition of MFSs. MetalS^2^ is described in the next section.

**Figure 2:**
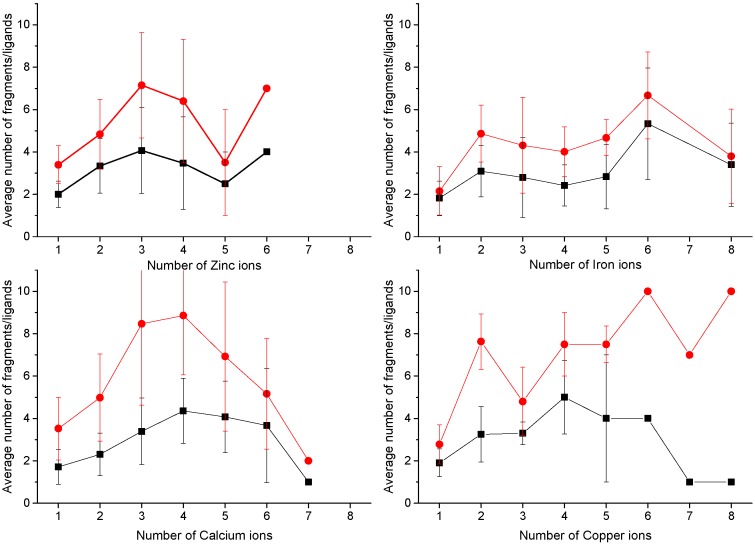
Average number of fragments (black lines and squares) and amino acid ligands (red lines and circles) as a function of MFS nuclearity for different metals. Only fragments containing at least one amino acid ligand to the metal ion(s) were counted. The statistics includes all MFS sites with a minimum number of donor atoms for each metal (three for zinc, four for iron and calcium, two for copper). This eliminates a significant part of adventitious sites at the protein surface. The data shown in this figure were mined from the public MetalPDB database [44], which is described in Section 3.

Figure 2 displays the distribution for different metals of the number of fragments containing at least one ligand to the metal fragments (black lines and points) and of the total number of metal ligands (red lines and points), as a function of the nuclearity (number of coupled metal ions) of the MFSs. There is a high variability of these values even within MFSs with the same number of ions bound for any given metal. Nevertheless, some trends are observed. In particular, the number of fragments increases with increasing nuclearity, up to four-ion sites (note that for all metals combined there are only about 30 MFSs with a nuclearity of five or more). This seems reasonable because the higher the nuclearity of MFSs, the larger their size. Therefore, they can recruit metal ligands from more distant parts of the protein. Iron is an exception due to the occurrence of iron-sulfur clusters, which are often coordinated by amino acids that are organized in groups close in sequence (for example, in many ferredoxins binding Fe_4_S_4_ clusters, three of the four cysteine ligands are within less than 20 sequence residues). Mononuclear iron and copper sites tend toward a smaller average number of ligands (2.1 and 2.8, respectively) than calcium and zinc sites (3.5 and 3.4, respectively). The reason for this is different for the two metals. The MFSs of copper(I) transporters feature two-coordination by the protein. If a non-protein metal ligand is recruited, then the coordination number can increase to three [38]. Instead, so-called type II copper sites can feature three-coordination by the protein, and again the coordination number can increase if there are additional exogenous ligands [39]. A larger number of metal ligands from the protein is present e.g., in type I mononuclear copper sites [40]. On the other hand, mononuclear iron sites are largely found in heme-containing proteins, where the porphyrin ring provides four donor atoms, and thus the protein occupies only one (e.g., in globins) or two (e.g., in most c-type cytochromes) coordination positions [41]. The protein provides a higher number of metal ligands in non-heme mononuclear sites, such as in enzymes like Fe-superoxide dismutase or in rubredoxin, the simplest iron-sulfur protein. Finally, as mentioned above, zinc(II) and calcium(II) MFSs have the same number of amino acid ligands. However, their coordination chemistry and preferences are significantly different, with zinc(II) in proteins preferring a total coordination number of four (also accepting three, five and six, but much less commonly than four) [42], and calcium(II) preferring a total coordination number of six and seven (five and eight are also accepted) [43]. The accidental high similarity in the number of ligands provided by the protein in calcium and zinc sites results from the fact that the former MFSs more often involve Glu/Asp side chains, owing to their harder chemical nature, each of which can occupy two coordination positions by acting as bidentate ligands.

**Figure 3:**
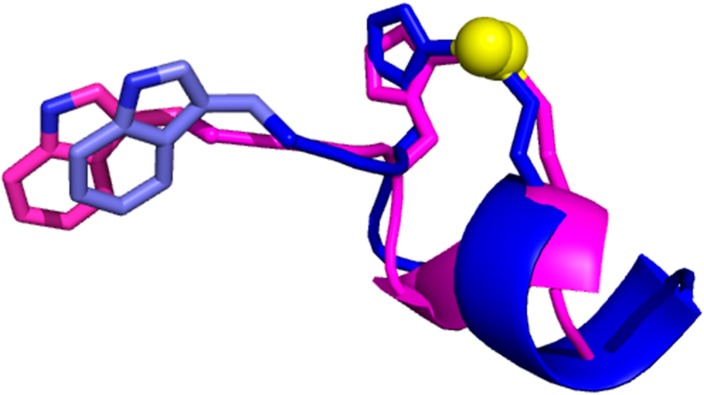
Structural superposition of two zinc-binding fragments containing the HX(2)C sub-pattern, found in PARP (**blue**) and PHD (**magenta**) zinc-finger domains. The Trp residues located in the second position before the His ligand (*i.e.*, WXHX(2)C) are shown as sticks. The zinc ions are shown as yellow spheres; the zinc-binding residues are shown as sticks.

There are multiple ways to group MFSs for their subsequent analysis. It is intuitive to group them by the identity of the bound metal ion(s) and/or by the chemical structure of the cofactor. For example, iron-binding MFSs can be separated into MFSs containing heme, iron-sulfur clusters, or individual iron ions. Among the latter, one can further discriminate MFSs binding a single ion or ligand-bridged multi-ion sites. Another option is to select MFSs that contain fragments sharing the same portion of MBP, *i.e.*, fragments containing a specific sub-pattern. These fragments can be aligned easily using the common sub-pattern as a seed. Then, statistics on the distribution of individual residues within the fragment can suggest the occurrence of interactions between metal ligands and neighboring residues in the sequence or the presence of catalytically important positions. For example, the alignment of zinc-binding fragments containing the HX(2)C sub-pattern highlights that the second position before the His residue is highly enriched in Trp, its frequency in this position being as high as 16 times the average frequency of Trp in proteins. The structural alignment of two fragments shown is taken from two different zinc-finger families, illustrating that this sequence pattern corresponds to a specific 3D motif (Figure 3). The present observation provides a rationale for the notion that sub-patterns are useful to improve the prediction of the metal-binding state of individual amino acids based on machine-learning approaches [31].

## 3. MetalPDB, a Database of Minimal Functional Sites in Metalloproteins

To enable analyses of the kind mentioned above, we created the MetalPDB resource [44], a database of MFSs derived from the structures deposited in the PDB. MetalPDB is available at http://metalweb.cerm.unifi.it. The crucial difference between databases of metal-binding sites and databases of ligands is the ready provision of information that is of specific interest to bioinorganic chemists. Metal ions interact with the macromolecular matrix via coordination bonds, defining the coordination geometry of the metal site (Figure 1). Databases of ligands do not compute or analyze this important feature even if they include metals and metal-containing cofactors in their content, because coordination geometry is not relevant for organic molecules [45,46]. An interesting resource is MetLigDB, a publicly accessible web-based database focused on the interaction between organic ligands and metals in in the active site of metalloproteins [47]. The scope of MetLigDB is significantly different from MetalPDB, which provides a metal-centered overview of all metal-binding biological macromolecules. The public database most similar to MetalPDB is MESPEUS [21]. MESPEUS focuses on the first coordination sphere of metal sites in metalloproteins of known 3D structure. MESPEUS also describes crystallographic features described extensively, and it permits the calculation of statistics for metals in any selected environment. With respect to MetalPDB, MESPEUS is providing greater geometric details but it is less useful to obtain biochemical information. For example, MESPEUS does not analyze the functional domains present in each metalloprotein and it does not categorize metal sites. These features are instead available in MetalPDB. Finally, the BioMe database allows the calculation of some properties of metal-binding sites [22], such as the frequency with which a selected amino acid appears in the coordination sphere of a given metal. The contents of BioMe partially overlap with the contents of MetalPDB.

MetalPDB can be searched using PDB IDs or keywords, or via an Advanced query interface. The database is automatically updated based on the procedure summarized below.

(1) All metal-containing structures released after the last update are downloaded from the PDB.
(2) For each metal ion in each structure from step (1) we identify the metal ligands, both within the polypeptide or polynucleotide chains (endogenous ligands) and different ions or molecules such as water, sulfide, acetate (exogenous ligands) (Figure 1B). Also organic cofactors such as heme are included in the exogenous ligands.
(3) Each pair of metal ions having at least one common ligand or being at a distance lower than 5 Å is included into a single dinuclear site. This procedure is iterated such that if metal A and metal B form a single site and then metal B and metal C also form a single site, eventually a trinuclear site is defined that contains all three metal ions. In this way, e.g., each Fe_4_S_4_ cluster found in ferredoxins constitutes an individual four-nuclear site.
(4) Identify the neighbors of all the metal ligands (both endogenous and exogenous) in each mono- or polynuclear site. Such neighbors are chemical species (residues in a polypeptide or a polynucleotide chain, or other molecules or ions) that contain at least one non-hydrogen atom at a distance smaller than 5 Å from the ligand itself. The ensemble of the neighbors, the ligands and the metal atom(s) constitute the MFS (Figure 1C).

The web interface of MetalPDB provides numerous pre-computed additional features for each MFS, such as coordination number and geometries or a list of hydrogen bonds involving metal ligands. MetalPDB systematically groups MFSs when they occur in the same position within a protein fold shared by various metalloprotein structures. In practice, for each metalloprotein the domain containing the ligands of the MFS is identified. Then pairs of proteins with a given domain (or having at least 50% sequence identity) are structurally superimposed, based on the fold of the domain of interest, and their MFSs are grouped if they occupy a corresponding position within the structure, as indicated by the distance between their geometric centers being less than 3.5 Å after fold superposition. We called the MFSs grouped according to this procedure “equistructural MFSs”. Equistructural MFSs are then split into groups of “equivalent MFSs”, within which all MFSs have the same nuclearity and contain the same metals. Thus, two equivalent MFSs are also equistructural, but the converse is not necessarily true. A single group of equistructural MFSs can correspond to one or more groups of equivalent sites [44]. Each group of equivalent sites in MetalPDB practically contains all the structures of the same metalloprotein that were independently solved as well as the structures of closely related proteins e.g., homologues from different organisms, provided they all bind the same metal ion(s). Thereby, they remove most of the degeneracy of the PDB contents and provide a meaningful way to analyze selected features of metalloproteins.

At present, MetalPDB contains more than 250,000 MFSs, extracted from more than 43,000 PDB entries. The most common metal is magnesium(II), which is present in 49.8% of the sites. Other common metals are zinc(II) (11.3% of all sites), calcium(II) (9.6% of all sites) and iron (7.9% of all sites). Note that the metal assignments of MetalPDB are automatically taken from the PDB, without any validation (see also the discussion of MetalS^3^ in Section 4).

**Figure 4:**
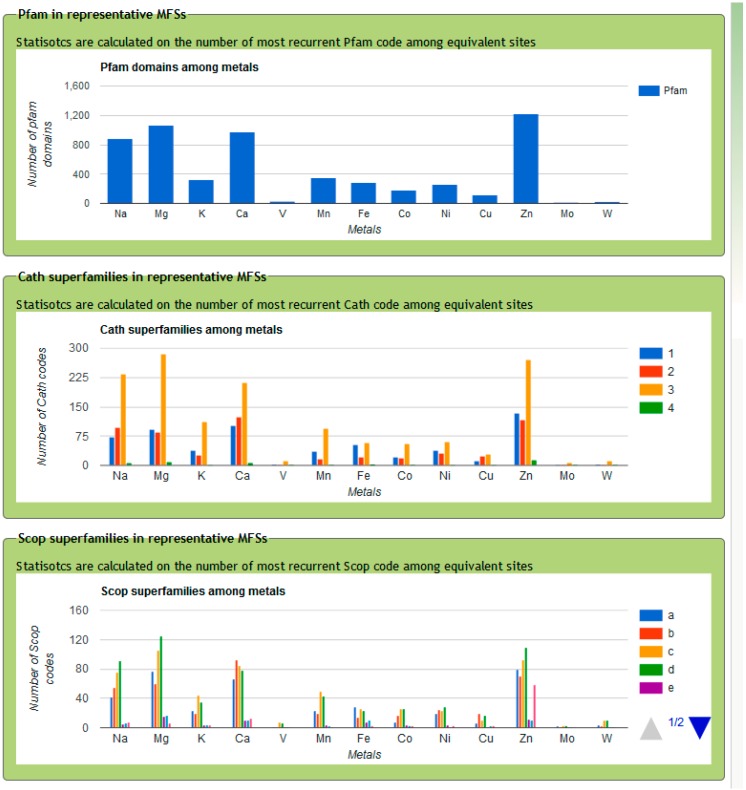
Number of different Pfam domains, CATH and SCOP superfamilies associated to each metal in MetalPDB. CATH and SCOP superfamilies are separated by Class (as an example, class 1 in CATH corresponds to mainly helical proteins).

As an example of analysis enabled by MetalPDB, Figure 4 shows the number of Pfam [48] domains and of different CATH [49] and SCOP [50] superfamilies associated to each metal. CATH and SCOP superfamilies are separated by their first level index (the protein class). One can readily appreciate that the majority of MFSs are in protein structures of the α/β CATH class (class 3) for all metals, whereas the relative abundance of MFSs in all-α (class 1) *vs.* all-β (class 2) structures is metal-dependent. For example, all-α structures are comparatively more common for iron. At the level of Pfam domains, zinc shows the greatest diversity, closely followed by magnesium, calcium and sodium.

## 4. MetalS^2^: A Tool for the 3D Structural Comparison of MFSs

The macromolecular frame around the metal ligands determines the chemico-physical properties and thus the reactivity of the metal ion(s) in the site. Consequently, MFSs can be structurally compares in a systematic manner in order to extract functional information for selected metal-binding macromolecules and/or entire metalloprotein families. To achieve this, we developed the MetalS^2^ software tool [51]. It is important to keep in mind that the overall structure of the macromolecules containing the sites does not affect the structural comparison of MFSs. Thus, the structural comparison of entire metalloproteins or of their MFSs only are two intrinsically complementary approaches [15,16].

The very first step of MetalS^2^ is to put the two metal sites at the center of the superposition. This crucial aspect differentiates our approach from any other approach to macromolecular structural comparison [51]. In practice, MetalS^2^ achieves this by overlapping the geometric centers of the metal ions in the two MFSs as the initial step. Then, each site is decomposed into an ensemble of units consisting of triangles whose vertices are the geometric center of the metal ions in the site and a pair of donor atoms. Thus, all such units share the first vertex. MetalS^2^ systematically overlaps all possible pairs of units from the two sites, always maintaining the vertices corresponding to the metal positions coincident [51]. The rationale of this procedure is to scan quickly for configurations (called “poses”) where the metal centers are coincident and the donor atoms overlap reasonably well. This first part of the MetalS^2^ algorithm is purely geometric and aims to ensure that the final superpositions will feature a good overlap of the first coordination sphere. All poses are ranked based on the MetalS^2^ quality function (“score”, see below). To evaluate the score it is necessary to define pairwise relationships between the atoms in the two MFSs. For this MetalS^2^ uses the Cα and Cβ atoms of proteins, and the N1 and N9 atoms of nucleic acids. Atoms are matched based on their distance. For each Cα atom from the first (query) site, we assign a correspondence to the Cα atom in the second (target) site that is closest in space. For any atom of the query site, MetalS^2^ restricts the search of a suitable correspondence to atoms of target site at a maximum distance of 2.0 Å. If no atom of the target structure falls in this range, no correspondence is created for the query atom. If both atoms in a Cα–Cα (or C1–C1) pair are bound to a Cβ (or N1/N9) atom, MetalS^2^ also computes the distance between the two Cβ atoms and associates them if their distance is below the threshold. Metal-binding residues are handled separately and can only be put in correspondence to metal-binding residues in the other MFS. To enhance coverage, a less restrictive threshold of 5.0 Å is used for metal ligands.

The MetalS^2^ score is defined as [51]:

(1) T=w1∑f=1F1nfN+ w2 ln(Cmaxc)+w3(1−SSmax)

with the three terms describing respectively:

1. the fragmentation of the alignment, by measuring how many fragments the alignment is broken into *(F*) and how long each fragment is (*n_f_*), *N* being the total alignment length
2. the relative coverage of the two sites, by comparing the total number of Cα and Cβ atoms in the shortest site (*C*_max_) to the number of atoms effectively put in correspondence (*c*)
3. the biochemical similarity of the residues put in correspondence, by comparing the BLOSUM62 similarity score (*S*) to the maximum possible score (*S*_max_).

The three weighting factors (*w*) have been defined empirically. The better the superposition, the lower the score of Equation (1) is. Optimal superpositions include a small number of long fragments, involve all atoms of the smallest MFS and put in correspondence atoms in the two sites that belong to the same or chemically similar residue type(s).

**Figure 5:**
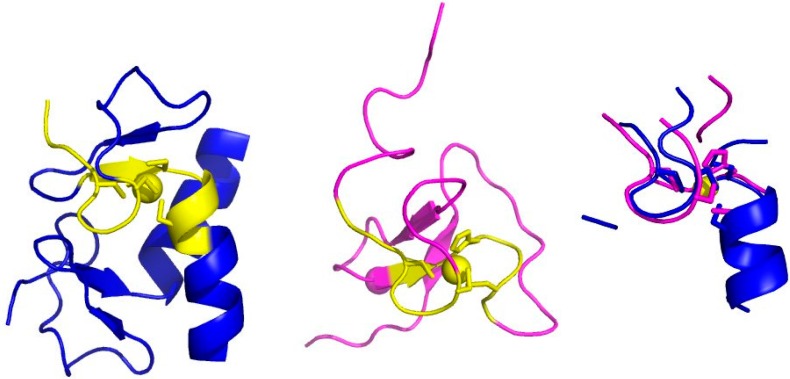
Overlap of two zinc MFSs embedded in two different folds. The full PDB structures clearly reveal different folds for the two proteins (PDB structures 1OVX, blue, and 1FP0, magenta), but the superposition of the two sites in the rightmost panel is very good (MetalS^2^ score = 2.29). The yellow regions in the two full structures correspond to the segments matched in the MFS superposition. The zinc ions are shown as yellow spheres; the zinc-binding residues are shown as sticks.

The poses that rank best in terms of their MetalS^2^ score are subject to a final optimization to maximize the structural superposition of the macromolecular parts of the MFSs, by minimizing the RMSD of the coordinates.

(2) $$RMSD=\sqrt{\sum_{i=1}^{C_{\text{max}}^{*}}\frac{{\left(x_{i}^{A}-x_{i}^{B}\right)}^{2}}{C_{\text{max}}^{*}}}$$

where *x_i_^A^* − *x_i_^B^* is the distance between the *i*-th atom pair, and C*_max_ is the number of matched Cα, Cβ atom pairs. The summation includes also the two metal ions of the two sites (or the virtual atoms corresponding to the geometric centers of polymetallic sites). The RMSD is minimized by roto-translating the target site. After roto-translation, the poses are re-ranked. The web interface of MetalS^2^ presents only the best scoring pose to the user. By running multiple benchmarks, we identified a threshold for the score of 2.75–3.0 for reasonable superpositions, for which however a manual inspection is appropriate, whereas scores of 2.0–2.25 or lower identify highly similar sites [15,51]. The MFS superpositions obtained with MetalS^2^ are completely independent of the overall protein fold, and indeed good superpositions can be achieved also for MFSs belonging to proteins with different folds (Figure 5).

The MetalS^3^ server uses an optimized version of the above algorithm to allow users to search the entire MetalPDB database for metal-binding sites that are structurally similar to the site of a structure of interest [15]. This input structure can be taken directly from the PDB or uploaded by the user. The latter scenario is useful to allow bioinorganic chemists to identify MFSs similar to metal sites discovered in newly determined 3D structures of metalloproteins. For a given input structure, the MetalS^3^ interface presents a table reporting the scores and superpositions of all the MFSs in MetalPDB that are structurally similar to the input metal-binding site. MetalS^3^ thus constitutes an unbiased approach to seeking structural similarities between metal-binding sites, independently of the user’s prior knowledge. Beyond the analysis of new sites, MetalS^3^ can help in the validation of metal assignments, by providing indications through the unbiased comparison to validated sites in the PDB. Indeed, the MetalS^3^ algorithm does not depend on the identity of the metal ion(s) present in the input MFS. Specialized tools for the assignment and validation of the identity of metal ions in crystallographic structures are also available [52,53]. A distinct advantage of these methods is their use of the experimental crystallographic data to support the output provided. A different task is the prediction of metal-binding sites from structural data of apo-proteins, *i.e.*, where the metal ion is undetectable or not bound to the site. Similarly to several tools used for the prediction of the binding of organic molecules to a receptor [54,55], many approaches for the prediction of metal-binding sites in 3D structures rely on the detection of conserved geometries involving potential metal ligands in the apo-protein structure, complemented by the use of machine-learning methods to rank the predictions [56,57,58]. We do not currently provide similar applications; however, the concept of MFS could be exploited in this direction, and possibly applied, upon appropriate re-definition, to the analysis and prediction of organic ligands.

**Figure 6:**
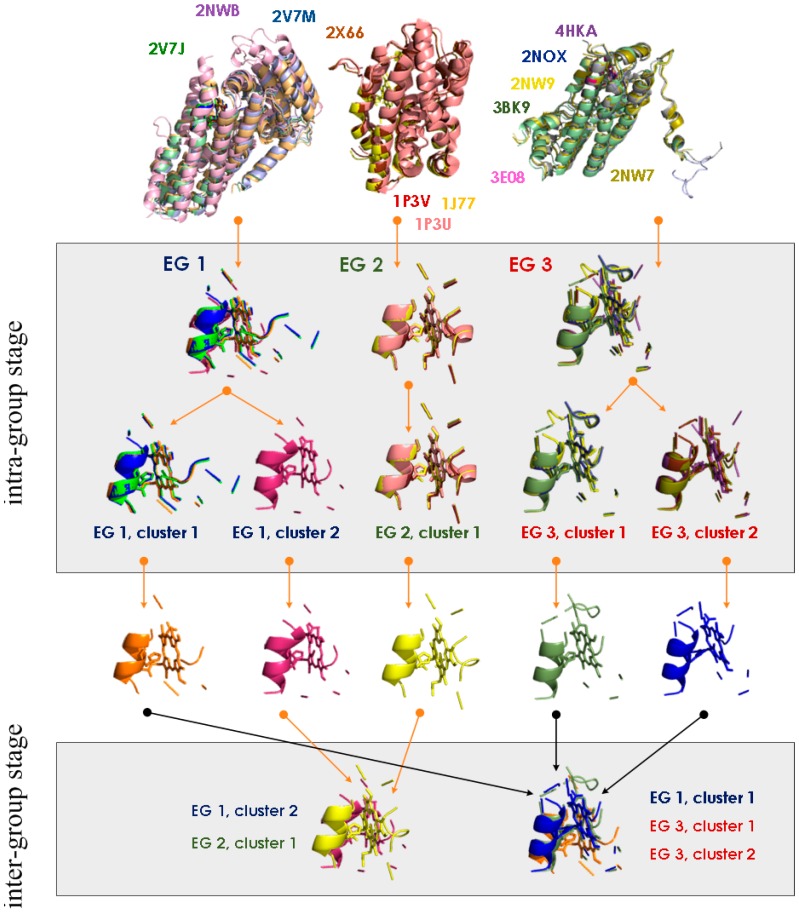
MetalS^2^-based clustering of heme-binding MFSs. Each protein structure and its MFS correspond to a different color in all panels. The PDB codes in the top panel show the color scheme used. All MFSs are extracted from the equistructural groups (EG) in the MetalPDB database that contain a heme-binding site (exemplified by EG 1, EG 2, EG 3 in the top row). The MFSs within each EG are clustered using a stringent threshold for the MetalS^2^ score (2.25 in the present example [16]). Consequently, MFSs from a given EG can be grouped in one or more clusters (EG1 and EG 3 give rise to two clusters each, whereas all MFSs in EG 2 are clustered together). This is the intra-group stage. Then, for each cluster a single representative is identified, as the MFS with the lowest cumulative distance (*i.e.*, sum of MetalS^2^ scores from all other MFSs in the cluster). The representative MFSs are finally clustered with a less stringent threshold and using a more relaxed hierarchical clustering approach (average rather than complete clustering). This is the inter-group stage. This clusters the MFSs regardless of the initial EG, thus putting together MFSs originally extracted from metalloproteins with different folds. This Figure was taken from [59].

The MetalS^2^ score allows MFSs to be organized systematically through a clustering approach [16]. This leverages the score as a quantitative measure of structural similarity between pairs of MFSs. We demonstrated the usefulness of this measure to build clusters of structurally similar MFSs using a two-stage hierarchical clustering algorithm. At the first stage, we cluster MFSs identified in corresponding position within proteins with the same fold, *i.e.*, included in the same equistructural group of the MetalPDB database. These clusters are created with a stringent threshold for the MetalS^2^ score (e.g., ≤2.25) and thus group only highly similar MFSs. This permits the meaningful definition of a single representative MFS for each cluster, which speeds up the entire procedure. Moreover, as we will describe later, the analysis of first-stage clusters permits the separation of each metalloprotein superfamily, characterized by a single common fold, into groups that are defined by the detailed structural features of the shared MFS. At the second stage of the clustering procedure, we compare and group all representative MFSs with a less stringent threshold (e.g., MetalS^2^ score ≤ 2.75) to detect similarities across different metalloprotein superfamilies. The resulting groups are thus independent of the overall protein fold. Figure 6 recapitulates the entire procedure.

**Figure 7:**
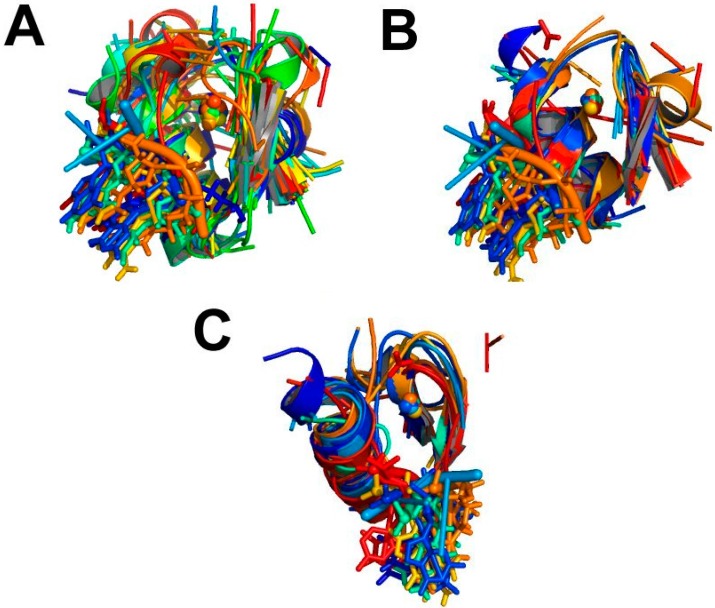
A cluster of representative zinc-binding MFSs containing 99 sites. Each MFS is assigned a different color (rainbow from 1 to 99), maintained in all panels. The zinc ions are shown as spheres. (**A**) Superposition of all 99 sites; (**B**) same as (**A**), but showing only the sites with an organic ligand bound; (**C**) a rotated top view of B.

As an example, the application of the procedure of Figure 6 to all the zinc sites in MetalPDB (contents of February 2015) produced 763 clusters of representative sites (inter-group stage in Figure 6) with more than one member [16]. Figure 7 shows a cluster of 99 sites from different zinc-finger and transcription factor families. The figure highlights the common location within the MFS of the DNA-recognition patch (Figure 7B,C), which can thus can be identified independently of global fold comparison.

**Figure 8:**
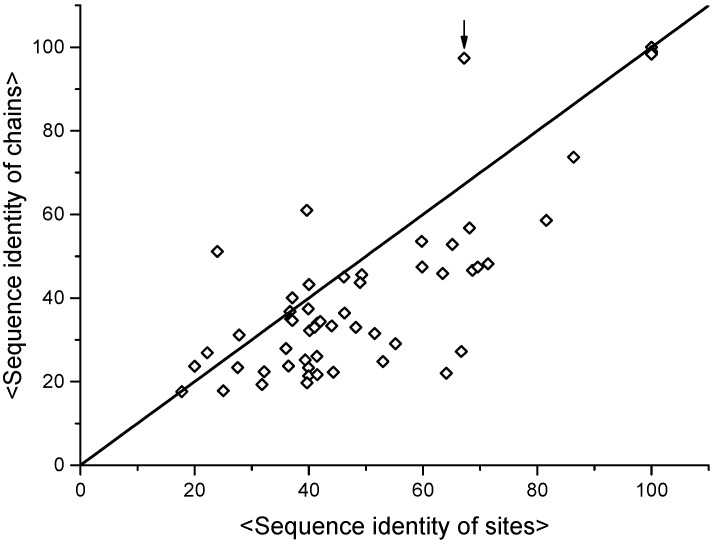
Comparison of sequence identity values within clusters of representative MFSs *vs.* sequence identity values of the corresponding protein chains. Heme-binding MFSs have been clustered using the approach of Figure 6. For each cluster, the pairwise sequence identity values between all sites have been computed from the structure-based alignment of the MFSs, and their average is reported on the *x* axis. The *y* axis instead reports the average sequence identity values obtained from the pairwise alignment of the protein chains containing the MFSs in each cluster. The continuous line corresponds to the function *y* = *x*; thus, points below the line correspond to clusters for which the average sequence identity of MFSs is higher than the average sequence identity of the proteins containing them. The point highlighted by the arrow corresponds to a cluster containing the MFSs from a single protein (multi-heme cytochrome *c*) with multiple repeats, plus another site. The *y* coordinate for this cluster is close to 100% because it includes the contribution due to the entire protein chain being aligned to itself.

Clusters of MFSs highlight common structural features across groups of sites in a fold-independent manner. These common features may underlie functional features as well, such as similar modes of interaction with substrates and cofactors (Figure 7). One may wonder if the observed conservation of structural features corresponds to local sequence similarity, at the MFS level. It is then instructive to compare sequence identity values within clusters of MFSs with respect to the sequence identity values obtained from the alignment of the corresponding entire protein chains. This is shown in Figure 8 for all clusters of heme-binding sites.

Figure 8 shows that aligned MFSs within clusters tend to have a larger similarity in sequence than the protein chains that contain them. The most prominent exception is given by multi-heme cytochromes *c*, a class of proteins that contain many individual heme-binding MFSs (up to more than 70 [60]). For such a system, the similarity between individual MFSs will always be lower than the chain similarity as the entire protein is actually compared to itself. For all heme-binding MFSs, there is on average an additional 10% in the sequence identity at the MFS level with respect to the comparison of entire protein chains. A paired-sample *t*-test suggests that this difference is meaningful at the 0.01 confidence level. This is a very interesting hint that evolution selects metal-binding sites by retaining local sequence features, which are presumably important for the proper local folding of the polypeptide chain, in spite of the divergence of the rest of the protein sequence. The points at the bottom right of the distribution in Figure 8 correspond to highly divergent protein sequences, with overall identity as low as about 20%, that share MFSs with highly similar sequences (up to 60%–70%). This validates the idea that local sequence similarity can be very informative for tasks such as the prediction of metal-binding capability from protein sequence [29]. It also lends support to the metalloprotein design approach where metal-binding sites sites are simply grafted onto well-defined protein scaffolds whose role is in practice mainly to allow proper folding of the grafted sequence [61,62]. The high conservation of MFS sequences in significantly divergent proteins indeed suggests that these sites can constitute independent units.

## 5. Concluding Remarks

Minimal functional sites in metalloproteins (MFSs) are portions of 3D structure that focus on the region around the metal site. This region is nearly always crucial for the physiological function of metalloproteins. Our analyses suggested that MFSs could recapitulate most of the functional properties of the proteins binding them, independently of their overall fold. MFSs thus enable a completely novel approach to the analysis of metalloproteins, which can complement the more traditional approaches based on global structural analysis.

In order to support the expansion of the MFS-based approach and its uptake by the community of bioinorganic chemists, we developed a number of software tools and protocols. Of particular relevance are: MetalPDB, a database automatically derived from the Protein Data Bank that contains all structurally characterized MFSs, and MetalS^2^, an algorithm to structurally compare pairs of MFSs in a quantitative manner. The availability of the latter similarity measure allowed us to cluster MFSs regardless of the global fold of the corresponding metalloproteins. In turn, this permits an independent assessment of the evolution of metal sites with respect to the evolution of the entire sequence of metalloproteins. A very interesting observation is that within each structural cluster the sequence of MFSs is less variable than that of the associated protein domains, in a statistically significant manner. Furthermore, MFSs similar in 3D structure and sequence can be identified within very divergent protein families, confirming the view that MFSs can be regarded as individual modules grafted onto the metalloprotein fold. Finally, MetalS^3^ allows users to compare a novel metal-binding site to all known MFSs available in MetalPDB, and thus has a unique potential to unveil unexpected relationships based on new 3D structure information.

## Abbreviations

MBP	Metal-binding pattern
MFS	Minimal Functional Site
PDB	Protein Data Bank
